# Bilateral carotid artery dissection due to Eagle syndrome in a patient with vascular Ehlers-Danlos syndrome: a case report

**DOI:** 10.1186/s12883-020-01866-2

**Published:** 2020-07-21

**Authors:** Hajime Ikenouchi, Masahito Takagi, Ayako Nishimura, Eriko Yamaguchi, Junpei Koge, Kozue Saito, Kazunori Toyoda, Masatoshi Koga

**Affiliations:** 1 Department of Cerebrovascular Medicine, National Cerebral and Cardiovascular Center, 6-1 Kishibe-shimmachi, Suita, Osaka, 564-8565 Japan; 2 Department of Neurology, National Cerebral and Cardiovascular Center, 6-1 Kishibe-shimmachi, Suita, Osaka, 564-8565 Japan; 3 Department of Neurology, Nara Medical University, 840 Shijo-cho, Kashihara, Nara, 634-8522 Japan

**Keywords:** Carotid artery dissection, Eagle syndrome, Ehlers–Danlos syndrome, Embolism, Transoral ultrasonography

## Abstract

**Background:**

Patients with vascular Ehlers-Danlos syndrome (EDS) occasionally suffer from arterial dissection. Eagle syndrome, which is caused by an elongated styloid process and also causes arterial dissection, is difficult to diagnose and could sometimes be overlooked. Little is known of the coexistence of these two diseases, and treatment strategy is not established. Here, we present a case of bilateral internal carotid artery (ICA) dissection due to Eagle syndrome in a patient with vascular EDS.

**Case presentation:**

A 30-year-old man was admitted to our hospital because of sudden onset of mild sensory disturbance in his left limbs. He had a history of Ehlers-Danlos syndrome (EDS) and also had left cervical internal carotid artery (ICA) dissection 3 years before. Diffusion-weighted imaging showed acute cerebral infarcts in the right hemisphere. Cervical computed tomography angiography (CTA) revealed the right ICA narrowing at the cervical portion in addition to the previous left cervical ICA dissection. Cervical magnetic resonance imaging (MRI) revealed double-lumen and intramural hematoma at the narrowing portion of the right cervical ICA, which indicates arterial dissection. CT also revealed bilateral elongated styloid processes which are close to each side of cervical ICA. We diagnosed him as bilateral ICA dissection due to bilateral Eagle syndrome. Considering vascular complications due to vascular EDS, we performed closer follow-up with transoral carotid ultrasonography (TOCU). In 4 months, his right ICA dissection gradually improved without stroke recurrence or deterioration of dissection.

**Conclusions:**

Since patients with vascular EDS easily develop arterial dissection, Eagle syndrome may be overlooked. Clinicians should consider Eagle syndrome in the case of vascular EDS with extracranial ICA dissection and close follow-up should be prioritized in cases of Eagle syndrome with vascular EDS.

## Background

Patients with vascular Ehlers-Danlos syndrome (EDS) occasionally suffer from vascular events including arterial dissection. Because of the high incidence of vascular complications, surgical treatments that include catheter intervention is controversial. Eagle syndrome, which is caused by an elongated styloid process and also causes arterial dissection, is difficult to diagnose and could sometimes be overlooked. Here, we present a case of bilateral internal carotid artery (ICA) dissection due to Eagle syndrome in a patient with vascular EDS.

## Case presentation

**Fig. 1:**
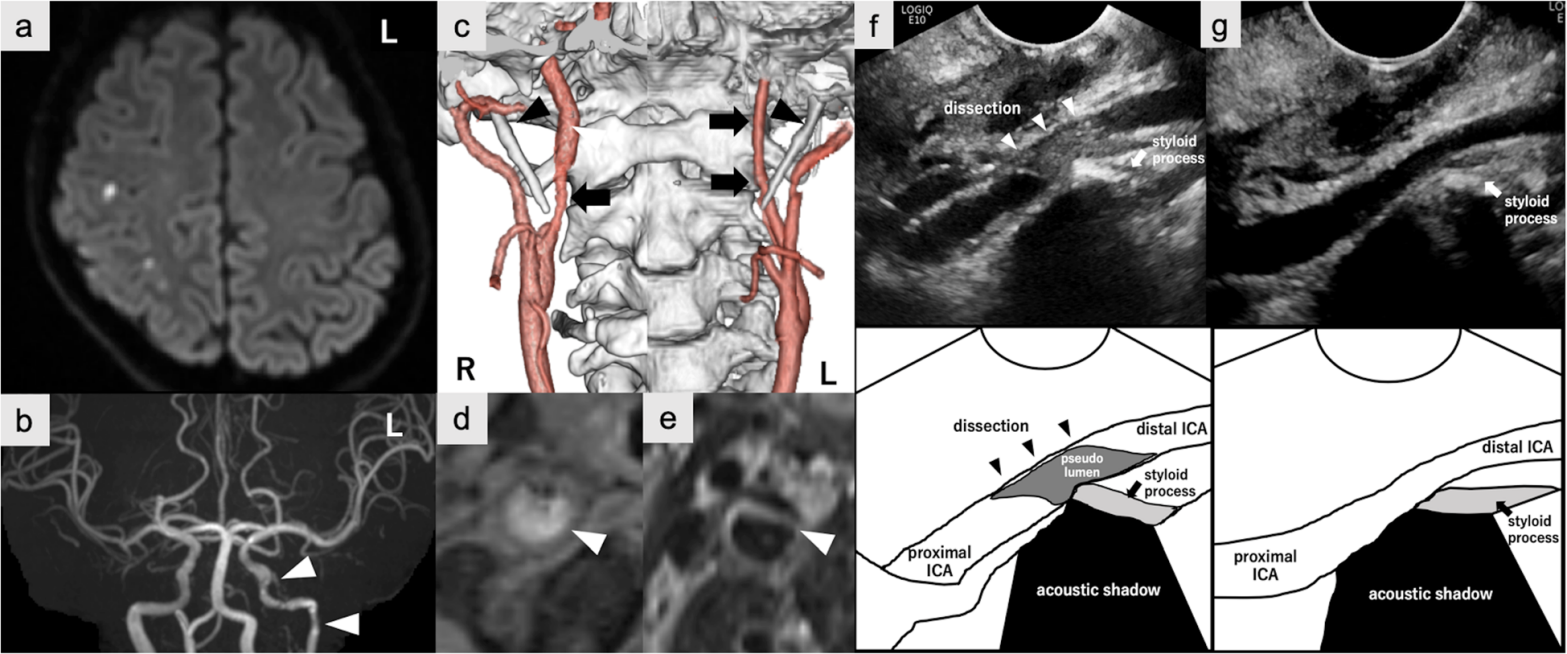
Diagnostic findings of bilateral ICA dissection and TOCU findings of right ICA dissection. **a**: Axial diffusion-weighted imaging showing multiple hyperintense signals in the right middle cerebral artery territory. **b**: Intracranial MRA showing no occlusion or stenoses in the right ICA territory. Multifocal segmental left ICA narrowing indicating previous arterial dissection *(arrowheads)*. **c**: Cervical CTA showing right extracranial ICA stenosis *(arrow)* and left ICA narrowing from the extracranial ICA *(arrows)*, accompanied by the bilateral elongated styloid process (*arrowheads*) close to each side of ICA. **d** and **e**: Cervical MRI showing intramural hematoma in T1-weighted imaging **d** and double-lumen in T2-weighted imaging **e**. **f**: Longitudinal view of TOCU showed right ICA stenosis with a thrombosed pseudolumen due to artery dissection (*arrowheads*). The styloid process (*arrow*) is spatially close to the dissection site accompanied by an acoustic shadow. g: Right ICA stenosis with a thrombosed pseudolumen decreased for 4 months of follow-up

A 30-year-old right-handed man was admitted to our hospital due to mild sensory disturbance in his left limbs. He had histories of sigmoid colon rupture and pneumothorax and underwent surgical therapies. Three years before, he was admitted with transient amaurosis due to left ICA dissection, and a gene mutation analysis revealed the presence of vascular EDS with a missense mutation of COL3A1 cDNA (c.1196 G > A). On current admission, he only had slight sensory disturbance and a National Institutes of Health Stroke Scale score of 1. There were no other neurological deficits including his cranial nerves. In brain magnetic resonance imaging (MRI), diffusion-weighted imaging showed multiple acute cerebral infarcts in the right middle cerebral artery territory (Fig. 1a). Intracranial magnetic resonance angiography (MRA) showed no occlusion or stenosis in the major arteries other than the known left ICA dissection (Fig. 1b). Cervical computed tomography angiography (CTA) depicted stenosis in the right extracranial ICA and left extracranial to intracranial ICA narrowing (Fig. 1c), accompanied by elongated styloid processes (right 3.5 cm, left 3.6 cm) spatially close to each side of the ICA (Fig. 1c). Cervical MRI revealed intramural hematoma and double-lumen in the right ICA (Figs. 1-1d and e). Transoral ultrasonography (TOCU) (LOGIQ E10, GE Healthcare) revealed severe stenosis with flow elevation due to a thrombosed pseudolumen and an elongated styloid process near the thrombosed pseudolumen (Figs. 1f). He was diagnosed with right ICA dissection due to vascular Eagle syndrome, which resulted in embolic stroke in the right hemisphere. Since his left ICA was narrowing from near the elongated styloid process, the left ICA dissection 3 y before was also caused by vascular Eagle syndrome. Considering his vascular vulnerability due to vascular EDS, surgical interventions were not performed. Instead, we performed secondary stroke prevention with aspirin and close follow-up with TOCU. In 4 months after the admission, his right ICA dissection improved without recurrence or deterioration of dissection (Figs. 1g) and aspirin was discontinued.

## Discussion and conclusion

This is a case of bilateral ICA dissection caused by bilateral Eagle syndrome in a patient with vascular EDS. There are several reports on arterial dissection in vascular EDS and those on arterial dissection in vascular Eagle syndrome; however, this is the first known case of coexisting vascular EDS and Eagle syndrome.

Vascular Eagle syndrome refers to a set of symptoms associated with an elongated styloid process, by which the cervical ICA is compressed, resulting in ICA dissection and cerebral infarction [1]. Although the incidence of an elongated styloid process itself is between 4 to 7%, the incidence of vascular Eagle syndrome is extremely rare [1], and clinicians occasionally misdiagnose it as idiopathic ICA dissection. Because ICA dissection caused by vascular Eagle syndrome has a higher recurrence rate than idiopathic ICA dissection [2], clinicians should consider vascular Eagle syndrome when diagnosing ICA dissection.

Treatment strategy was controversial because there was no case of coexisting Eagle syndrome and vascular EDS. In cases of carotid artery dissection due to Eagle syndrome, either surgical resection of the elongated styloid process or carotid artery stenting are sometimes conducted [2]. On the other hand, in patients with vascular EDS, the surgical intervention is thought to be harmful or sometimes life-threatening because of the vascular vulnerability, instead that arterial dissection is a well-known vascular complication among them [3]. In our patient, left ICA dissection did not recur or deteriorate for 3 y. This suggests ICA dissection due to Eagle syndrome in vascular EDS could be safely observed without surgical intervention. Since half of the cases of arterial dissection caused by Eagle syndrome did not recur or deteriorate without surgical intervention [2], close follow-up should be prioritized in the coexistence of Eagle syndrome and vascular EDS, and surgical intervention should be restricted only in vascular EDS cases with the life-threatening situation [4, 5].

In conclusion, Eagle syndrome should be considered as an important causative condition of extracranial ICA dissection even in vascular EDS patients. Close follow-up should be prioritized in cases of Eagle syndrome with vascular EDS.

## Data Availability

Not applicable.

## Abbreviations

EDS: Ehlers-Danlos syndrome
ICA: internal carotid artery
CTA: computed tomography angiography
MRI: magnetic resonance imaging
MRA: magnetic resonance angiography
TOCU: transoral carotid ultrasonography
